# Blocking of targeted microRNAs from next-generation sequencing libraries

**DOI:** 10.1093/nar/gkv724

**Published:** 2015-07-23

**Authors:** Brian S. Roberts, Andrew A. Hardigan, Marie K. Kirby, Meredith B. Fitz-Gerald, C. Mel Wilcox, Robert P. Kimberly, Richard M. Myers

**Affiliations:** 1 HudsonAlpha Institute for Biotechnology, Huntsville, AL 35806, USA; 2 Department of Genetics, University of Alabama at Birmingham, Birmingham, AL 35294, USA; 3 Center for Clinical and Translational Science, University of Alabama at Birmingham, Birmingham, AL 35294, USA; 4 Department of Medicine, Division of Gastroenterology and Hepatology, University of Alabama at Birmingham, Birmingham, AL 35294, USA; 5 Department of Medicine, Division of Clinical Immunology and Rheumatology, University of Alabama at Birmingham, Birmingham, AL 35294, USA

## Abstract

Highly abundant microRNAs (miRNAs) in small RNA sequencing libraries make it difficult to obtain efficient measurements of more lowly expressed species. We present a new method that allows for the selective blocking of specific, abundant miRNAs during preparation of sequencing libraries. This technique is specific with little off-target effects and has no impact on the reproducibility of the measurement of non-targeted species. In human plasma samples, we demonstrate that blocking of highly abundant hsa-miR-16–5p leads to improved detection of lowly expressed miRNAs and more precise measurement of differential expression overall. Furthermore, we establish the ability to target a second abundant miRNA and to multiplex the blocking of two miRNAs simultaneously. For small RNA sequencing, this technique could fill a similar role as do ribosomal or globin removal technologies in messenger RNA sequencing.

## INTRODUCTION

The measurement of small RNAs, and in particular microRNAs (miRNAs), is an important tool in many biological fields due to their key roles in fundamental cellular processes such as cell fate maintenance, DNA damage response, cell-cycle regulation and others (reviewed in (1–3)). miRNAs primarily function as modulators of specific mRNA levels by targeting various protein complexes (RISC and others) to mRNA through complementary base pairing (reviewed in (4)). Additional functions of miRNAs have also been reported (5,6). Aberrant expression of miRNAs is linked to numerous human diseases, including cancer, cardiovascular disease, neurodegenerative disease and obesity (7–10). Importantly, the discovery of stable miRNAs in readily accessible human biological fluids such as blood and urine has increased the potential utility of miRNAs as practical biomarkers for these diseases (11–25).

Small RNAs can be measured with a variety of technologies, including qPCR, microarrays and solution-based hybridization, amongst others. Next-generation DNA sequencing (NGS) is also a powerful method for the discovery and quantification of small RNAs due to its technical performance (26), low expense, ultra-high throughput and its ability to agnostically detect and measure new species. However, NGS of small RNAs has several technical challenges. Among these is the well-reported biased behavior of the modified forms of T4 RNA Ligase 2 commonly used in sequencing library generation protocols. This bias manifests in small RNA libraries as differential ligation, creating an over-representation of certain species and an under-representation of others (27–31). When small RNA libraries are constructed from many sample types, these biases in ligation efficiency, combined with inherent abundance differences, can yield inaccurate results. Highly abundant small RNA species may be preferentially ligated such that their representation in the library becomes inordinately high, diminishing the ability to measure other more lowly abundant species. The precise detection of these lowly represented species would thus require very high sequencing depths and proportionally higher costs. Additionally, highly abundant species interfere with many normalization techniques, limiting the utility of the collected reads.

In small RNA libraries made from human plasma and serum, many of the most highly abundant species are probably derived from blood cell populations (32,33). While these may be of interest in some applications, miRNAs and other small RNAs that act as biomarkers for many diseases, such as cancer and neurodegenerative disease, may be of low abundance in the blood of afflicted patients (34–36). Accordingly, the problem facing researchers interested in blood-based miRNA biomarkers is how to measure precisely low-abundance species in a background of highly abundant and less informative species that comprise most of the reads in sequencing library.

Here we present a method whereby highly abundant species can be eliminated from a small RNA library prior to sequencing. We demonstrate in human plasma samples that this reduction leads to an increase in the total number of miRNAs detected without compromising the reproducibility of the measurement of abundance or the ability to detect differential expression of miRNAs. We also establish that multiple species can be eliminated simultaneously without unwanted effects.

## MATERIALS AND METHODS

### Total RNA isolation

The protocol for collection of peripheral blood samples was approved by the Institutional Review Board at the University of Alabama at Birmingham, and all donors provided written, informed consent. Blood was collected into EDTA-tubes. Within 30 min of collection, the plasma was isolated (∼5 ml) and stored at −80°C. One milliliter plasma was centrifuged at 14 000 relative centrifugal force for 15 min and total RNA was isolated from the supernatant using the Plasma/Serum Circulating and Exosomal RNA Purification Kit (Slurry Format) (Norgen Biotek) following the manufacturer's directions. The eluate from this kit was further concentrated using the RNA Clean-Up and Concentration Kit (Norgen Biotek) using 20 μl elution buffer to collect the RNA.

### Small RNA sequencing and miRNA blocking

Isolated total RNA containing miRNA was converted to cDNA sequencing libraries according to the method described in Vigneault *et al*. (2012) and Eminaga *et al*. (2013), with modification (our full protocol can be found in Supplemental Methods). Briefly, for each library, 4 μl isolated RNA was combined with one μl of 10 μl 3′ adaptor and 1 μl T4 RNA Ligase 2, truncated (NEB) in the appropriate buffer for 1 h. Simultaneously, 1 μl 0.5 μM miRNA blocking oligonucleotide was incubated for 5 min at each of the following temperatures: 95°C, 65°C, 55°C, 45°C and 35°C to ensure the proper formation of the hairpin structure. Next, incubated blocking oligonucleotide was as added to the 3′ adaptor ligation product and incubated for 1 h at 30°C and 15 min at 65°C in the presence of T4 DNA Ligase (NEB) in the appropriate buffer to anneal and block the targeted miRNA from further reactions. One microliter of 10 μM reverse transcription primer was annealed to the 3′ adaptor ligation product for 5 min at 75°C, 30 min at 37°C and 15 min at 25°C prior to the addition of the 5′ adaptor in order to reduce formation of adaptor-dimer products. One microliter of 20 μM pooled 5′ adaptor was incubated for 2 min at 70°C and then ligated with T4 RNA Ligase 1 (NEB) to each reaction product for 1 h at 25°C. Ligated reaction products were reverse transcribed using SuperScript II (Invitrogen) and amplified via PCR using Phusion High-Fidelity PCR Master Mix (NEB). The thermal cycling conditions were 94°C for 30 s, followed by 15 cycles of 94°C for 10 s and 72°C for 45 s and a final extension at 65°C for 5 min.

Libraries were cleaned and concentrated using a MinElute PCR Purification Kit (Qiagen), following the manufacturer's instructions, and eluted into a final volume of 20 μl. Libraries were separated on a TBE-Urea 10% acrylamide gel (Bio-Rad) with warm buffer for 50 min. The band corresponding to miRNAs (∼135–145 base pairs) was excised, eluted from the gel, precipitated and resuspended in 10 μl of EB Buffer (Qiagen). Small RNA library concentration was quantified by the Library Quantification Kit - Illumina/ABI Prism (KAPA Biosystems) and sequenced on a HiSeq2000 or a MiSeq according to standard Illumina protocols.

### Data processing and analysis

Adaptor sequences were trimmed from the raw fastq files using Cutadapt (37). The trimmed reads were aligned to pre-miRNA sequences (miRBase version 19) (38) using Bowtie2 (39). The alignments were filtered to keep only those alignments that had two or fewer base mismatches and yielded a unique best alignment as measured by the Bowtie2 alignment score. The remaining unaligned reads were then aligned to the hg19 reference genome using Bowtie2. Again, unique best reads were required. For miRNAs, read counts were obtained by counting the overlaps of the reads aligned to the pre-miRNAs with the canonical mature form boundaries (miRBase version 19) using BEDtools (40). Any overlap with the mature region was counted.

The miRNA read counts for each experiment were down-sampled to a common level using random sampling implemented in R (base package). When the ‘average’ of two replicates was taken (generally for plotting), the following procedure was used: the two libraries were down-sampled to a common total count value and then counts for each species were summed. This summed library was then down-sampled to the original common total count value. We favor this process for averaging replicate libraries because it preserves the count nature of the data and accordingly the underlying distribution. Differential expression was calculated using the package DESeq2 (41) in R using ‘local’ dispersion estimates and ‘LRT’ tests. A significant result was defined as one with Benjamini-Hochberg adjusted *P*-value <0.01. Dispersion estimates were calculated with DESeq2 as well using the ‘local’ mode. Prior to plotting in Figure 4A, the estimates were smoothed using the spline function in R (base package).

## RESULTS

### Blocking hsa-miR-16–5p in sequencing libraries

In a set of small RNA sequencing libraries from 27 human plasma samples that we prepared by using a slightly modified version of the protocol described by Alon *et al*. (28), we observed that reads mapping to hsa-miR-16–5p comprised between 20 and 60% of the total aligned reads in the libraries (Supplementary Figure S1). Furthermore, consistent with other reports (32,33), hsa-miR-16–5p levels correlated with the degree of hemolysis present in the sample. The massive abundance of hsa-miR-16–5p in these libraries makes sequencing to a sufficient depth to detect lowly abundant miRNAs very expensive. Proper normalization of libraries in which one or few species dominate the reads is problematic. Also, because the hsa-miR-16–5p level varies, sequencing multiple samples to a common depth, in terms of non-hsa-miR-16–5p reads, is difficult.

**Figure 1.**
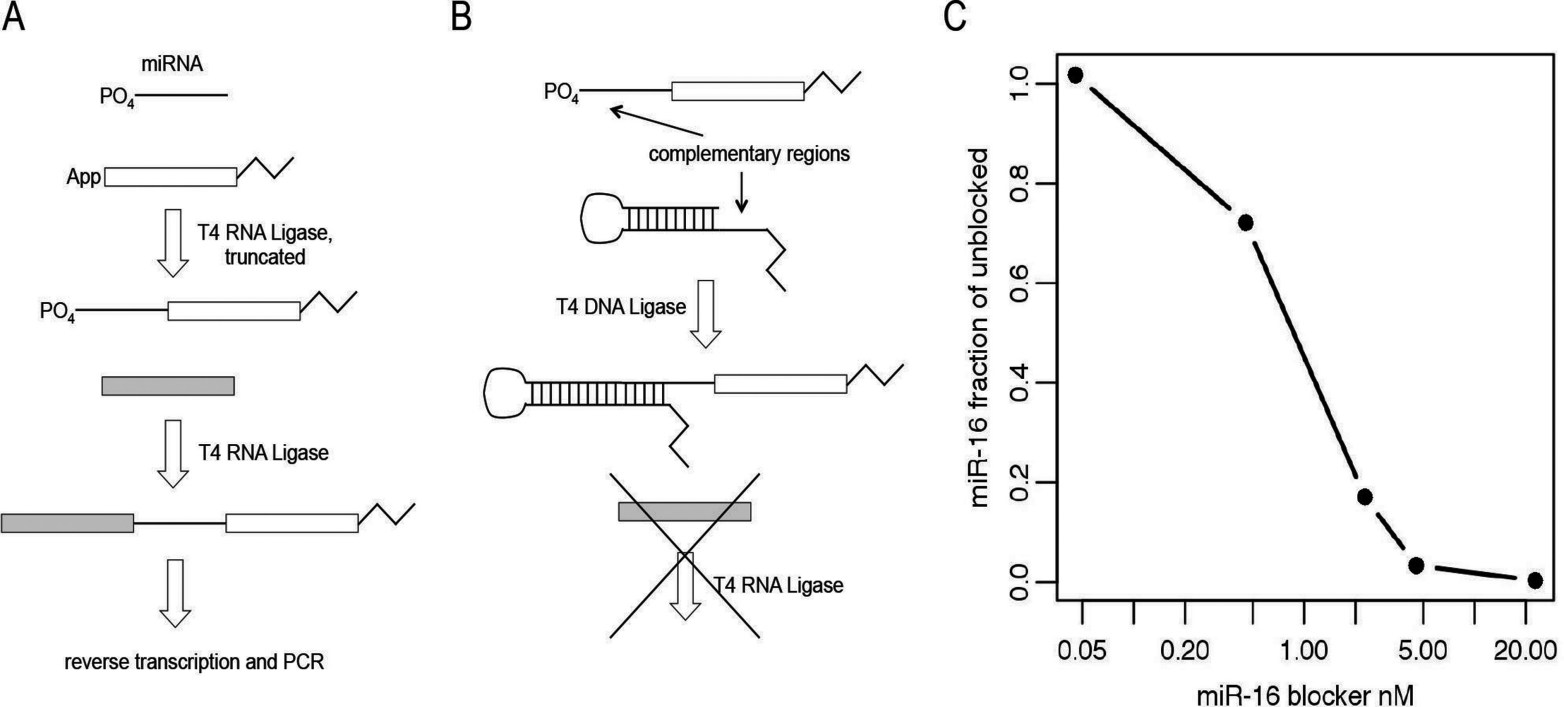
Modification of miRNA sequencing library generation protocol to allow for blocking of targeted species. (**A**) In the standard protocol, a pre-adenylated adaptor is ligated to the 3′ end of a small RNA pool using T4 RNA Ligase 2, truncated. Subsequently, a second adaptor is added to the 5′ end of the miRNA with T4 RNA Ligase 1, followed by reverse transcription and PCR. (**B**) In our modification, a hairpin oligonucleotide with an overhang complementary to the 5′ end of the targeted miRNA is attached via ligation with T4 DNA Ligase to the 5′ end of the miRNA subsequent to the ligation of the adaptor to the 3′ end. This prevents the ligation of the second adaptor to the 5′ end of the miRNA, resulting in a product that does not amplify during PCR. (**C**) Sequencing libraries were generated from human heart total RNA using a titration of a blocking oligonucleotide targeting hsa-miR-16–5p. The fraction of hsa-miR-16–5p present in the blocked library compared to the unblocked library is shown on the y-axis.

To resolve these issues, we devised an approach to remove hsa-miR-16–5p from the sequencing libraries by blocking it as a substrate of T4 RNA Ligase 1 during the ligation of the adaptor to the 5′ end (Figure 1). In the standard protocol (Figure 1A), a pre-adenylated DNA oligonucleotide adaptor is ligated to the 3′ ends of the pool of small RNA species using truncated T4 RNA Ligase 2. Subsequently, a RNA oligonucleotide adaptor is ligated to the 5′ ends using unmodified T4 RNA Ligase 1. The resulting product is reverse transcribed and amplified with PCR. In our modified protocol (Figure 1B), we use an oligonucleotide comprised of a self-complementary hairpin with a 12-base overhang on its 5′ end that is the reverse complement of the first 12 bases of the 5′ end of the canonical sequence of the targeted miRNA. The 5′ end of the oligonucleotide is modified with a C3 spacer (propyl group) to prohibit its participation in any unwanted ligation reactions. This ‘blocker’ oligonucleotide is introduced after the ligation of the pre-adenylated adaptor to the 3′ ends of the small RNA pool but prior to the ligation of the adaptor to the 5′ ends. The complementary portions of the targeted miRNA species and the blocker participate in Watson-Crick base pairing to form a double-stranded RNA:DNA hybrid with a missing phosphodiester bond between the 3′ end of the blocker and the 5′ end of the targeted miRNA, comprising a ‘nick’. T4 DNA Ligase recognizes this hybrid molecule and seals the nick (NEB product literature), resulting in the blocker being covalently bound to the 5′ end of the target miRNA. The presence of the hairpin and the C3 blocker prevent the subsequent ligation of the adaptor to the 5′ end of this product. Without the primer binding sequence contained in the adaptor, this ‘blocked’ product is not amplified in downstream PCR, effectively removing it from the final library.

To demonstrate the efficacy of this approach, we titrated various concentrations of a blocker targeting hsa-miR-16–5p into library generation reactions using human heart total RNA as the input. Human heart total RNA is a suitable test sample since hsa-miR-16–5p is abundant in libraries derived from it, comprising ∼10% of the miRNA reads. The effect on hsa-miR-16–5p read abundances in the final sequenced libraries shows dose-response behavior (Figure 1C), with a maximal effect in the 5–20 nM range. Furthermore, we applied this blocking method by using the hsa-miR-16–5p blocking oligonucleotide at 20 nM in a set of libraries derived from 23 human plasma samples. In the sequenced libraries, hsa-miR-16–5p was reduced to <1% of the reads in all cases (Supplementary Figure S2), far lower than in the previous set without blocking (Supplementary Figure S1).

Because we anticipated that targeting miRNAs at their 5′ ends would lead to off-target activity due to sequence homology within miRNA families, we initially attempted to target and block miRNAs from the 3′ end. Analogous to the 5′ approach, we used a hairpin oligonucleotide with a complementary 3′ overhang, a 5′ phosphate and 3′ C3 blocker. The blocking ligation with T4 DNA Ligase occurs first, before the ligation of the adaptor to the 3′ ends of the small RNA pool. Although this approach did effectively block hsa-miR-16–5p in human heart total RNA (data not shown), it had an adverse effect on the final library yields. In fact, even in libraries subjected to a mock blocking ligation reaction that included all reagents except the blocker oligonucleotide, this 3′ approach yielded final library concentrations approximately five times lower than the 5′ approach (Supplementary Figure S3). This decrease in yield in the 3′ approach is likely due to the leftover ATP from the initial blocking ligation with T4 DNA Ligase inhibiting the truncated T4 RNA Ligase 2 in the subsequent ligation of the adaptor to the 3′ ends of the small RNA pool. Although truncated T4 RNA Ligase 2 cannot turnover ATP, ATP can still bind to the remnants of the active site, leading to inhibition of the enzyme (personal communication with NEB). Thus, a 3′ approach could likely be implemented without unwanted consequences if the reaction components of the blocking ligation were removed via column purification or some other suitable method. However, the fractional recovery of the small RNA from these methods can be low. Considering the intended application of this method to human plasma samples in which the RNA concentrations are already low, further reduction of the effective RNA input is undesirable. Furthermore, miRNAs are known to have considerable variation at their 3′ ends due to differences in Dicer cut sites and non-templated nucleotide additions (42,43). Because our approach relies on T4 DNA Ligase, which is sensitive to base-pair mismatches and gaps (44), these variations can adversely affect the efficacy of the blocking (Supplementary Figure S4). Although variation at the 5′ end has similar effects on the 5′ blocking approach (Supplementary Figure S5), 5′ end variants generally represent a smaller fraction of the total. Considering these limitations of the 3′ approach, we decided to focus on the 5′ approach for further studies.

### Evaluating the quantitative performance of blocked libraries

To rigorously evaluate the effect of blocking hsa-miR-16–5p on the measurement of the non-targeted miRNA species in the library, we generated libraries from five human plasma samples. For each sample, we generated two libraries that were unblocked, that is, subjected to the blocking ligation reaction without a blocking oligonucleotide. Additionally, we generated two libraries using a blocking oligonucleotide targeting hsa-miR-16–5p, for a total of four libraries per sample. We chose the plasma samples to have a high degree of hemolysis such that the blocking of hsa-miR-16–5p should have large effects.

**Figure 2.**
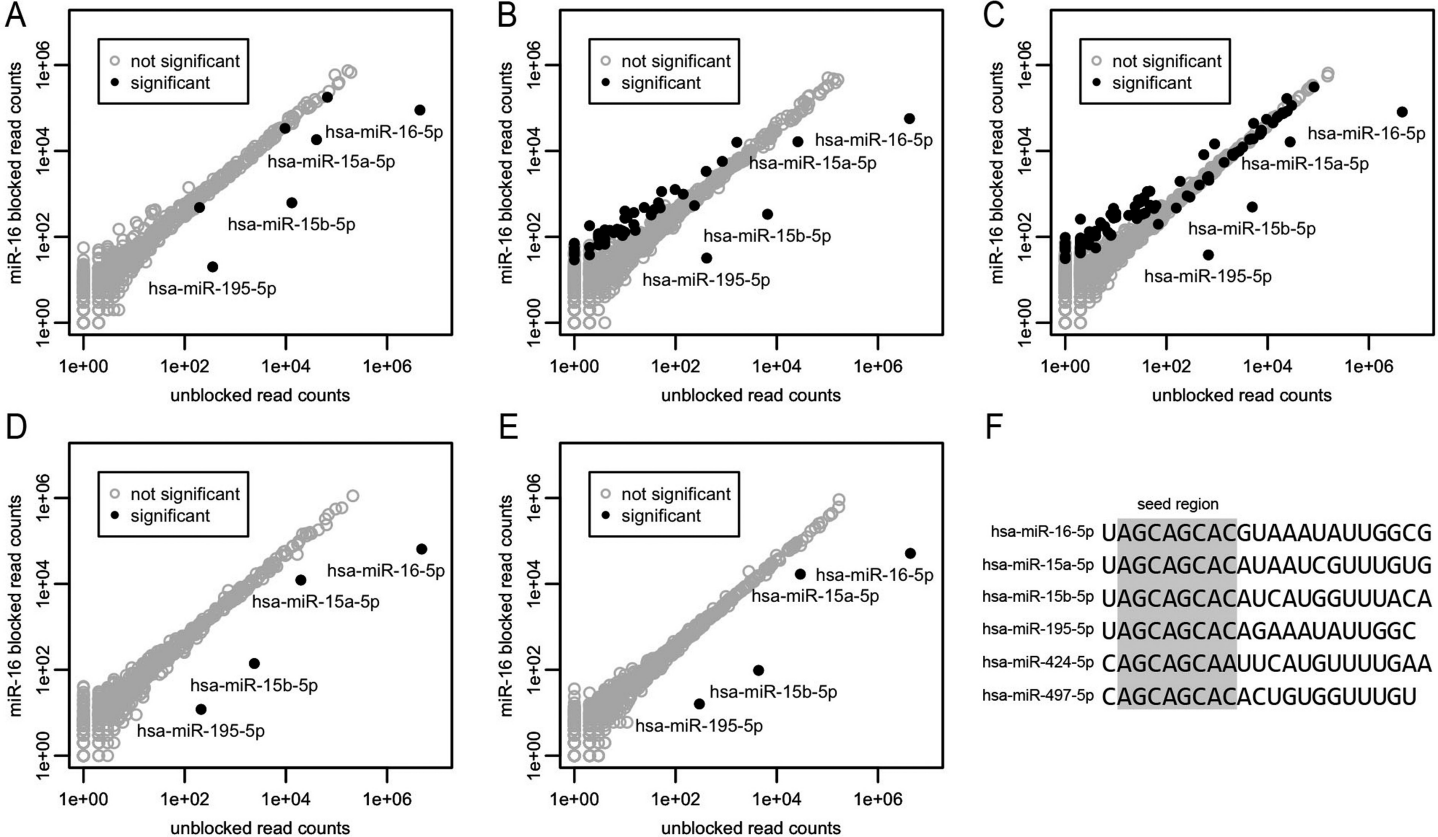
Blocking of hsa-miR-16–5p in human plasma samples. (**A–C**) Sequencing results from five different human plasma samples are shown in A–E. Read counts from averaged (see Materials and Methods) replicate unblocked and hsa-miR-16–5p blocked libraries are shown on the x and y axes respectively. All libraries were down-sampled to 6 million aligned miRNA reads before plotting and analysis. A miRNA is considered significantly differentially expressed between the two conditions if the adjusted *P*-value as calculated by DESeq2 is <0.01. Not significantly differentially expressed miRNAs are shown as open gray circles. Significantly differentially expressed miRNAs are shown as filled black circles. (**F**) Sequences of the mir-16 family members are shown with the seed region (bases 2–8) highlighted in gray.

For each sample, the replicate unblocked and hsa-miR-16–5p blocked libraries were analyzed by using the application DESeq2 (see Materials and Methods) to establish those miRNAs differentially affected by the blocking. The analysis was limited to miRNA species alone because the accurate alignment of non-miRNA species was not universally precise enough to allow for meaningful comparisons. As expected, members of the mir-16 family are also blocked by this approach, due to sequence similarity at their 5′ ends (Figure 2A–E). Interestingly, mir-16 family members hsa-miR-424–5p and hsa-miR-497–5p are not blocked, likely because they have a cytosine in the first position rather than the uracil that the other four members have (Figure 2F). This is consistent with the inability of T4 DNA Ligase to seal nicks at positions where a base pair mismatch is present at the nick site (44). We observed that in two of the five samples, a small number of non-targeted miRNAs are significantly lower in the blocked libraries as well (Figure 2A–C). Some of these miRNAs have several bases of sequence similarity with the blocker oligonucleotide and would form a duplex with a one-base gap between its 5′ end and the 3′ end of the blocking oligonucleotide. The inefficiency in sealing these gaps explains the moderate fold-changes. Other lowered species have no obvious sequence similarity. They are only significantly lower in one sample (Figure 2C) and have small fold changes, suggesting that possibly the FDR correction used by DESeq2 did not sufficiently correct the multiple hypothesis effect. Several miRNAs are actually significantly higher in the blocked libraries (Figure 2B and C). Presumably, these miRNAs were able to be more effectively ligated during 5′ adaptor ligation in the absence of the highly abundant and preferred ligation substrate hsa-miR-16–5p.

**Figure 3.**
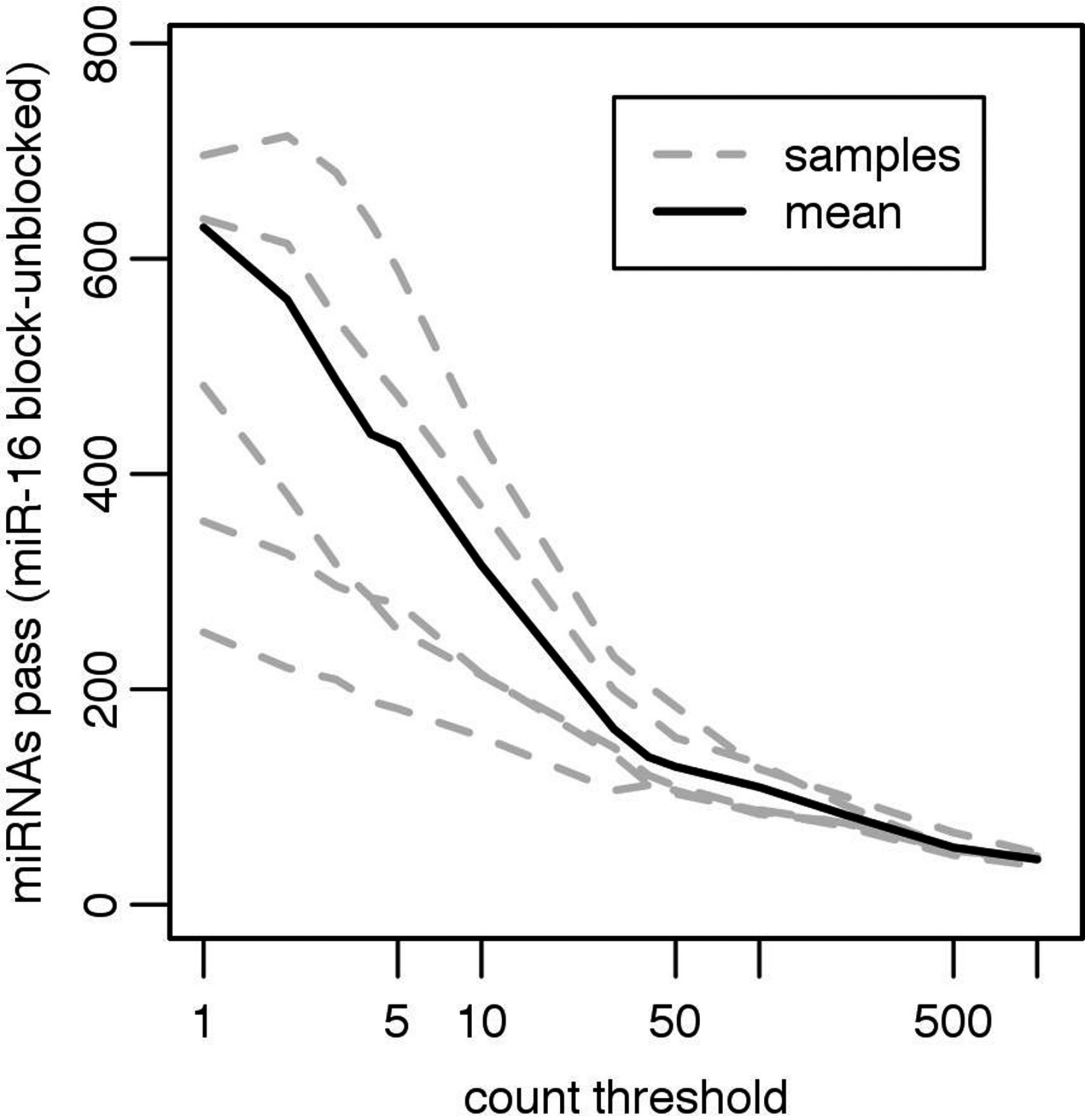
Effect of hsa-miR-16–5p blocking on read depth in human plasma samples. A set of count thresholds is plotted on the x-axis versus the difference between the number of miRNAs passing that threshold in the hsa-miR-16–5p blocked samples versus the unblocked samples is plotted on the y-axis. The differences between individual samples are shown as gray dashed lines. The mean difference is shown as a solid black line. All libraries were down-sampled to 6 million aligned miRNA reads before plotting.

An important motivation for blocking hsa-miR-16–5p in these samples was to increase detection of the low-abundance species. With a basis of an equivalent number of aligned reads, comparison of the unblocked libraries to the blocked libraries shows a marked increase in the number of miRNA species detected at a variety of count thresholds in all five plasma samples (Figure 3). At a commonly chosen cutoff of 10 counts, between 180 and 450 more miRNAs are detected at this threshold in blocked samples compared to unblocked samples. This improvement in the detection of the low-abundance species was accomplished with negligible increase in library generation costs and no increase in sequencing costs.

A critical concern is that the blocking protocol adversely impacts the reproducibility of the measurement of non-targeted miRNAs and by extension, the ability to precisely measure differential expression. While it is reassuring that the measured abundances of the vast majority of miRNAs are not affected by the blocking protocol (Figure 2), it is important to note that given the known bias caused by the RNA ligases used in the library generation protocol, the absolute abundance of the miRNAs in the library does not represent a strictly meaningful measurement of the actual abundance in the sample. Nevertheless, the goal of many studies is to measure differential expression between sample groups. In these cases, the abundance of miRNAs needs only to be measured reproducibly.

**Figure 4.**
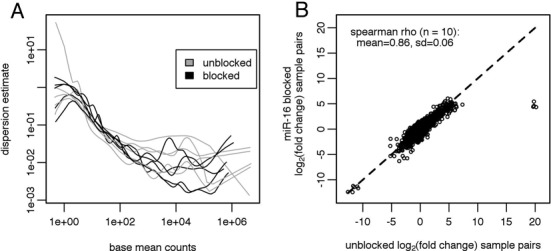
Effect of hsa-miR-16–5p blocking on reproducibility and differential expression measurement in human plasma samples. (**A**) Dispersions were calculated for each set of plasma sample libraries based on the replicate unblocked libraries and replicate hsa-miR-16–5p blocked libraries using DESeq2. The dispersion values are plotted on the y-axis versus the base mean read counts, also calculated by DESeq2, on the x-axis. The unblocked dispersions are plotted in gray while the blocked dispersions are plotted in black. (**B**) Fold changes were calculated between all possible sample pairs (10) in both unblocked and hsa-miR-16–5p libraries. The log_2_(fold changes) for all of those pairs are plotted on the same axes, with the log_2_(fold change) for the unblocked library on the x-axis and the log2(fold change) for the hsa-miR-16–5p blocked library on the y-axis. Thus each point represents a unique miRNA-sample pair combination. Only those miRNAs for which both samples had a DESeq2-calculated base mean >10 were plotted. The mean and standard deviation of the set of 10 Spearman rhos of the correlation of the fold changes between unblocked and hsa-miR-16–5p blocked libraries is listed on the plot.

To assess the reproducibility of the five human plasma sample libraries, we calculated the Spearman rho coefficient of correlation between replicate libraries (Supplementary Figure S6). For all five samples, the Spearman rho was higher for the blocked. However we are concerned that correlation may not be the best measure of reproducibility in these libraries because the biases introduced by the RNA ligases are consistent. Thus, correlation may be imposed upon a set of two libraries simply because they were subjected to the same bias. As an alternative, we used DESeq2 to estimate the dispersions of each library based on its replicates (see Materials and Methods). DESeq2 proposes a negative binomial distribution as the appropriate distribution for count data (41) and estimates the dispersion as function of read depth. As seen in our data, the dispersion is generally highest at low counts and decreases with increasing read depth (Figure 4A). The blocked libraries show no greater dispersion in any count regime, and may be less dispersed, particularly in the middle-to-high count range.

Lastly, for blood-based biomarker studies, the ability to measure differential expression is paramount. Using DESeq2, we calculated the fold changes between all possible pairs of samples (10 pairs) separately in both the unblocked libraries and the hsa-miR-16–5p blocked libraries. The measurement of the fold change of miRNAs between two samples is highly similar in the unblocked and hsa-miR-16–5p blocked libraries (Figure 4B). With the exception of a few outliers, the vast majority of fold changes scatter around the unity slope line (dashed line in Figure 4B). We calculated the Spearman coefficient of correlation between the unblocked and hsa-miR-16–5p blocked libraries for each of the 10 pairs. The coefficient values were very high, with a mean of 0.86 (Figure 4B). These data indicate that the blocking of hsa-miR-16–5p has very little effect on the measurement of differential expression in this sample set.

### Extension of the blocking technique to other miRNAs and multiplexing

**Figure 5.**
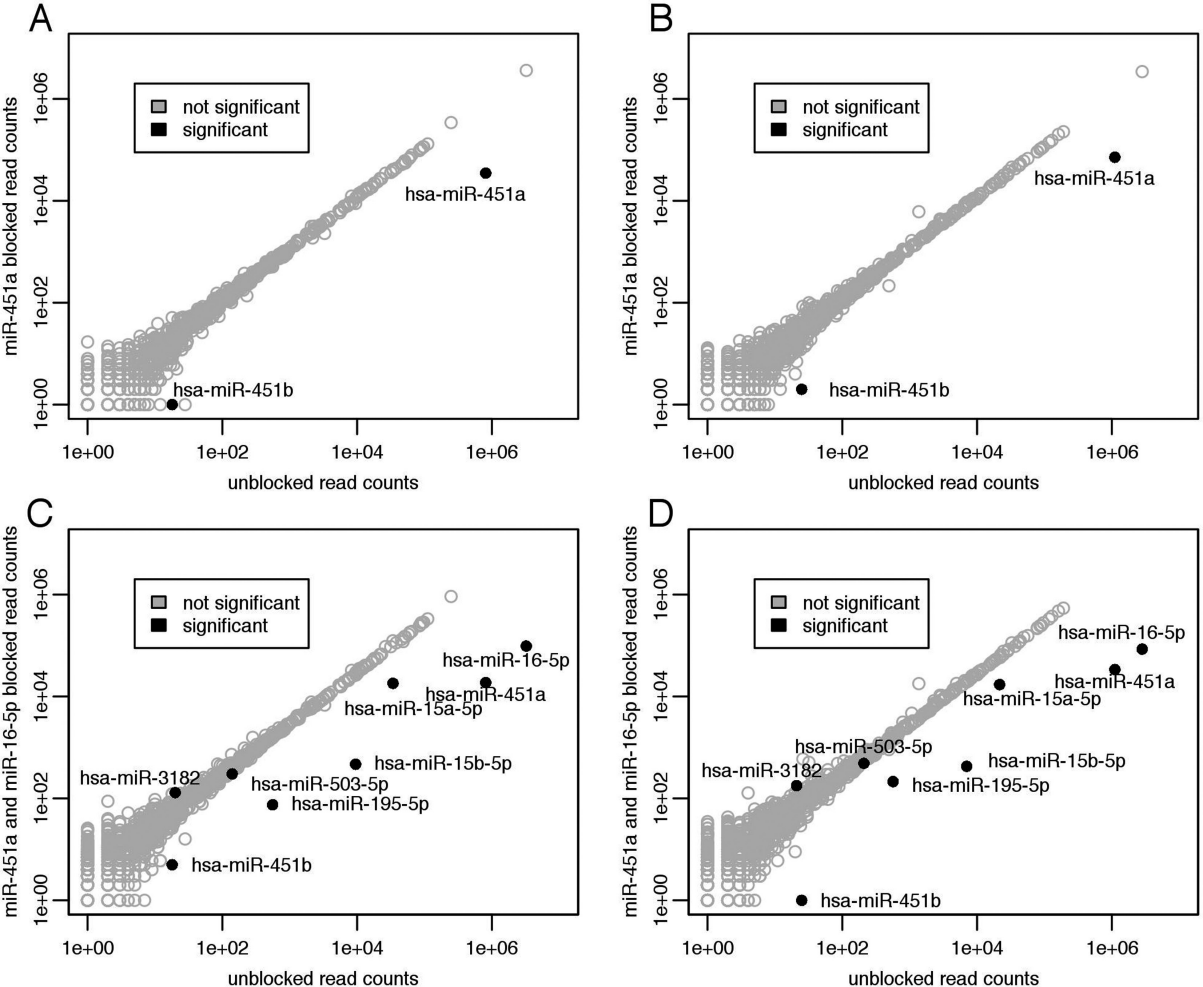
Blocking of hsa-miR-451a alone and in concert with blocking hsa-miR-16–5p in human plasma samples. (**A–B**) Sequencing results from two human plasma samples are shown. Read counts from an unblocked library and a hsa-miR-451a blocked library are shown on the x and y axes respectively. (**C–D**) Sequencing results from the same two human plasma samples are shown. Read counts from an unblocked library and a hsa-miR-451a and hsa-miR-16–5p simultaneously blocked library are shown on the x and y axes respectively. A miRNA is considered significantly differentially expressed between the two conditions if the adjusted *P*-value as calculated by DESeq2 is <0.01 and if its base mean count is above 50. Not significantly differentially expressed miRNAs are shown as open gray circles. Significantly differentially expressed miRNAs are shown as filled black circles. All libraries were down-sampled to 6 million aligned miRNA reads before plotting and analysis.

To establish the ability of the blocking method to block species other than hsa-miR-16–5p, we blocked hsa-miR-451a with an appropriately designed blocker oligonucleotide in a set of two human plasma samples. We chose hsa-miR-451a because it is also abundant in our libraries prepared from human plasma samples and because it has been implicated to be derived from blood cells, like hsa-miR-16–5p (32). Our analysis found hsa-miR-451a to be effectively blocked by this approach with minimal off-target effects (Figure 5A and B). Other than the intended target, hsa-miR-451a, only hsa-miR-451b was significantly affected by the blocking. Although the canonical mature form of hsa-miR-451b lacks significant sequence similarity to hsa-miR-451a, we found that the reads mapping to the hsa-mir-451b hairpin actually aligned near its stem-loop portion and do show sequence similarity with the 5′ portion of the hsa-miR-451a canonical mature form.

The ability to combine blocking oligonucleotides in a single blocking reaction would allow for reduction of a chosen set of miRNAs. We combined blocking oligonucleotides targeting hsa-miR-16–5p and hsa-miR-451a into a single blocking reaction. The combination resulted in both blocking oligonucleotides behaving as they did when in isolation (Figure 5C and D). Other than the miRNAs expected to change based on the single blocker experiments, only hsa-miR-503–5p was significantly down regulated, albeit with a small fold-change. Hsa-miR-503–5p shares seven bases of identical sequence with hsa-miR-16–5p on the 5′ end. The combination of blocking oligonucleotides had no discernible effect on the total library yield. Overall, combining multiple blocking oligonucleotides seems to be a viable strategy. The extent to which blocking oligonucleotides can be multiplexed is the subject of future research.

## DISCUSSION

Highly abundant and likely marginally informative miRNAs in NGS datasets from human serum or plasma hinder one's ability to discover true small RNA species functioning as biomarkers. We have ameliorated this problem by demonstrating a method to block miRNAs from representation in sequencing libraries. This method uses inexpensive reagents and requires no additional clean-up steps. Application of the method in human plasma samples resulted in a robust blocking of hsa-miR-16–5p, an abundant blood cell contaminant.

As a result of this blocking, the read depth of low-abundance miRNAs was dramatically increased, leading to the detection of a greater number of species and a more accurate measurement of differential expression. Off-target effects do occur based on sequence homology at the targeted end of the miRNA, in this case the 5′ end, especially within miRNA family members. However, these off-target effects are limited and predictable. The method does not decrease the reproducibility of the measurement of non-targeted miRNAs and has no ill effects on the measurement of differential expression.

We generalize the approach by targeting a second miRNA, hsa-miR-451a. Again, the performance of the blocking method on hsa-miR-451a is specific and has very small effects on non-targeted species. Additionally, the combination of two blocking oligonucleotides targeted to hsa-miR-16–5p and hsa-miR-451a in one blocking ligation reaction produced the same results seen by each one separately and without any interaction effects. This result implies the ability to combine several blocking oligonucleotides into a single reaction, although it remains to be tested.

We anticipate that this technology could fill a role in small RNA sequencing similar to that which ribosomal RNA and globin RNA reduction methods have in messenger RNA sequencing. Although our research focused on small RNA sequencing in human plasma samples, the method could be useful in other tissue types as well. Custom pools of blocking oligonucleotides could be tailored to a particular application to maximize the use of sequencing resources. Also, even though our experimentation focused on the use of the Illumina platform, we expect this method to be applicable to other platforms as long as the library generation method relies on the ligation of adaptors directly to small RNAs. When it is anticipated that the small RNAs of interest will be rare and lowly expressed, as is likely true in many applications, our method offers a robust and cost-effective way to precisely measure them.

## Supplementary Material

SUPPLEMENTARY DATA
