# Supplementary material for: Blocking of targeted microRNAs from next-generation sequencing libraries
Source: Nucleic Acids Res. 2015 Jul 23;43(21):e145. doi: 10.1093/nar/gkv724 (PMC4666382; doi:10.1093/nar/gkv724)
Supplement: SUPPLEMENTARY DATA [file supp_43_21_e145__index.html]

Blocking of targeted microRNAs from next-generation sequencing libraries — Blocking of targeted microRNAs from next-generation sequencing libraries — SUPPLEMENTARY DATA 

# Blocking of targeted microRNAs from next-generation sequencing libraries

## SUPPLEMENTARY DATA

- SUPPLEMENTARY DATA
