# Supplementary material for: Blocking of targeted microRNAs from next-generation sequencing libraries
Source: Nucleic Acids Res. 2015 Jul 23;43(21):e145. doi: 10.1093/nar/gkv724 (PMC4666382; doi:10.1093/nar/gkv724)
Supplement: SUPPLEMENTARY DATA [file supp_gkv724_nar-01082-met-k-2015-File007.pdf]

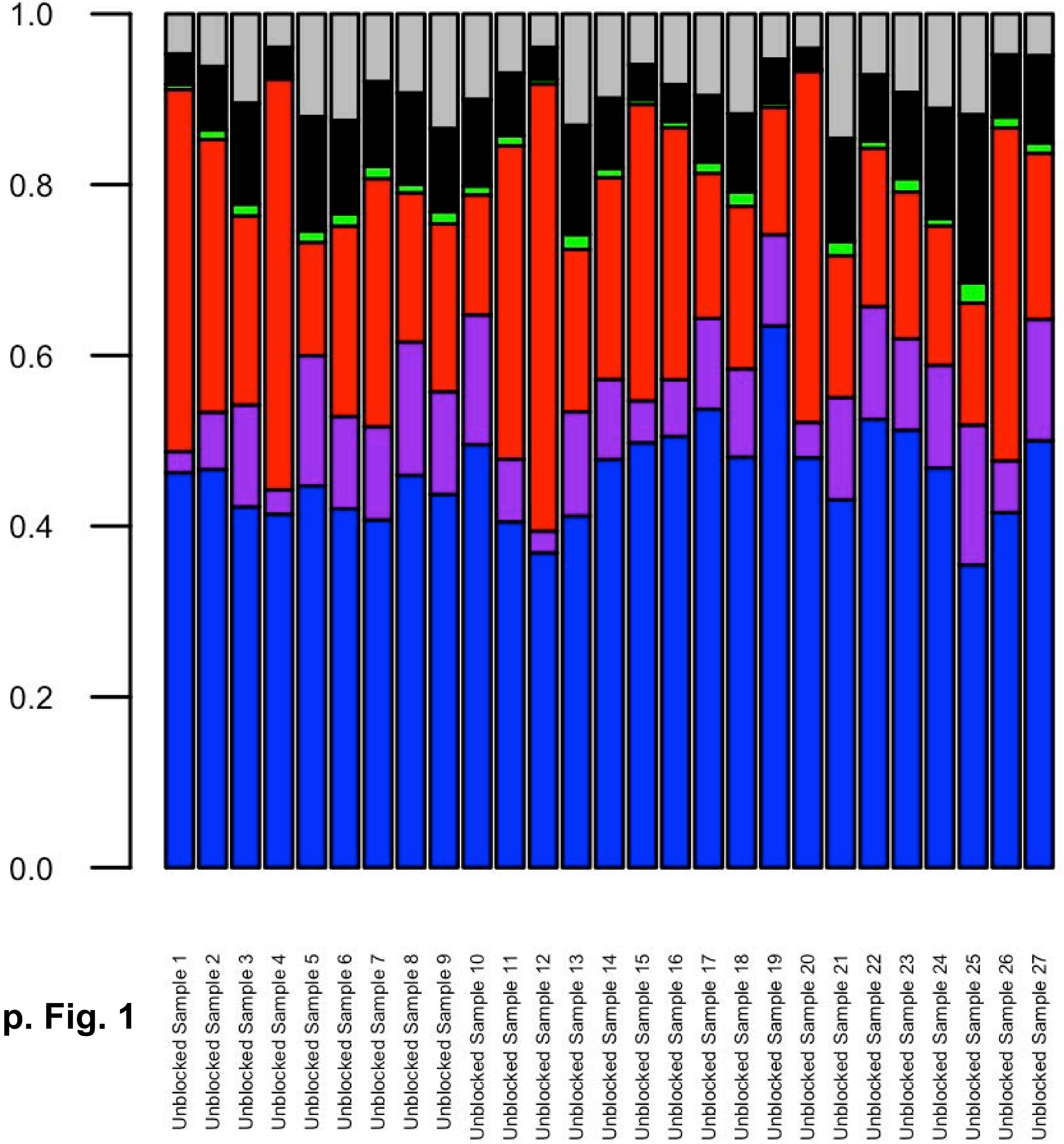
1.0
0.8
0.6
0.4
0.2
0.0
Unblocked Sample 1
Unblocked Sample 2
Unblocked Sample 3
Unblocked Sample 4
Unblocked Sample 5
Unblocked Sample 6
Unblocked Sample 7
Unblocked Sample 8
Unblocked Sample 9
Unblocked Sample 10
Unblocked Sample 11
Unblocked Sample 12
Unblocked Sample 13
Unblocked Sample 14
Unblocked Sample 15
Unblocked Sample 16
Unblocked Sample 17
Unblocked Sample 18
Unblocked Sample 19
Unblocked Sample 20
Unblocked Sample 21
Unblocked Sample 22
Unblocked Sample 23
Unblocked Sample 24
Unblocked Sample 25
Unblocked Sample 26
Unblocked Sample 27

Sup. Fig. 1

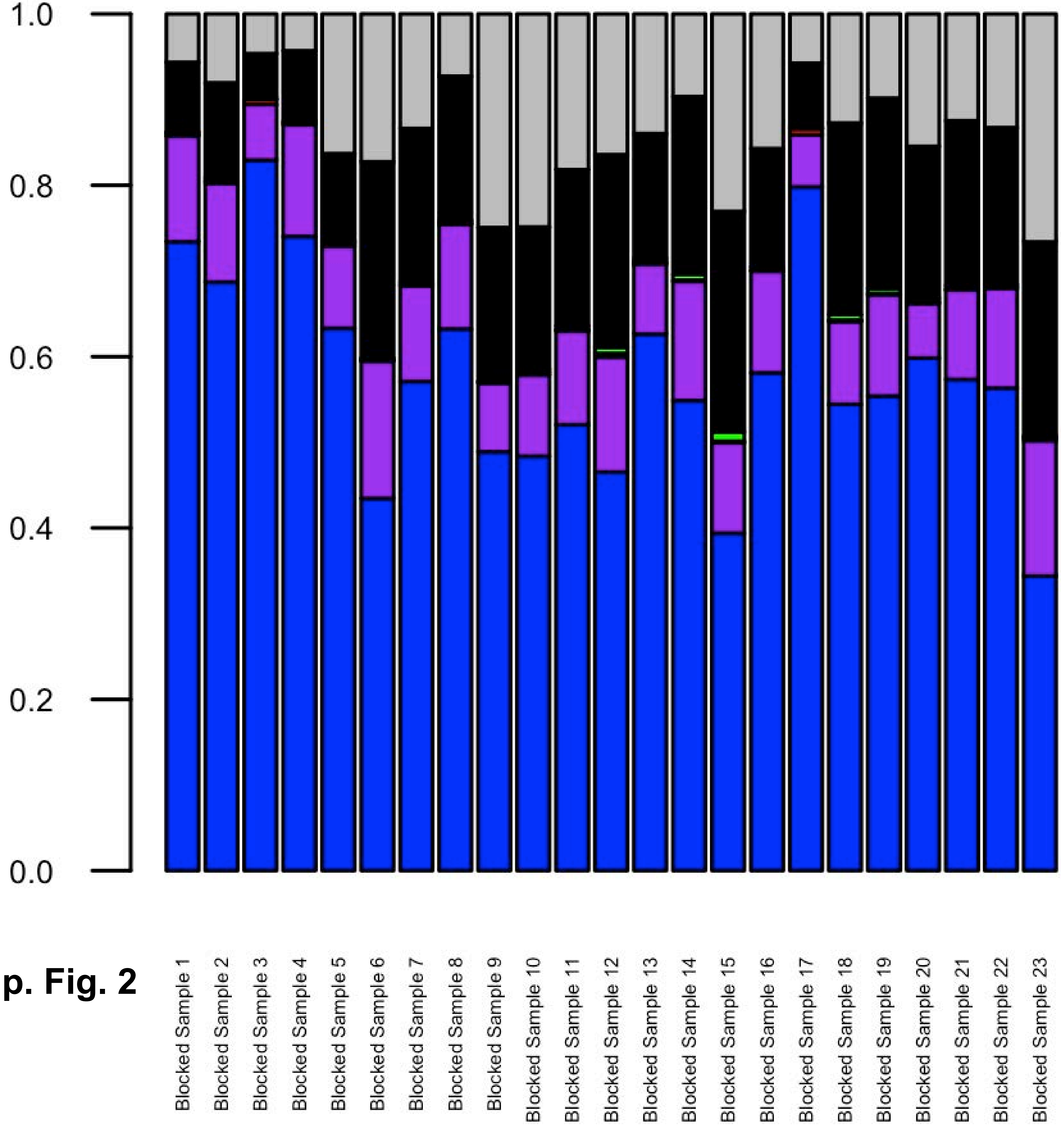
1.0
0.8
0.6
0.4
0.2
0.0
Blocked Sample 1
Blocked Sample 2
Blocked Sample 3
Blocked Sample 4
Blocked Sample 5
Blocked Sample 6
Blocked Sample 7
Blocked Sample 8
Blocked Sample 9
Blocked Sample 10
Blocked Sample 11
Blocked Sample 12
Blocked Sample 13
Blocked Sample 14
Blocked Sample 15
Blocked Sample 16
Blocked Sample 17
Blocked Sample 18
Blocked Sample 19
Blocked Sample 20
Blocked Sample 21
Blocked Sample 22
Blocked Sample 23

Sup. Fig. 2

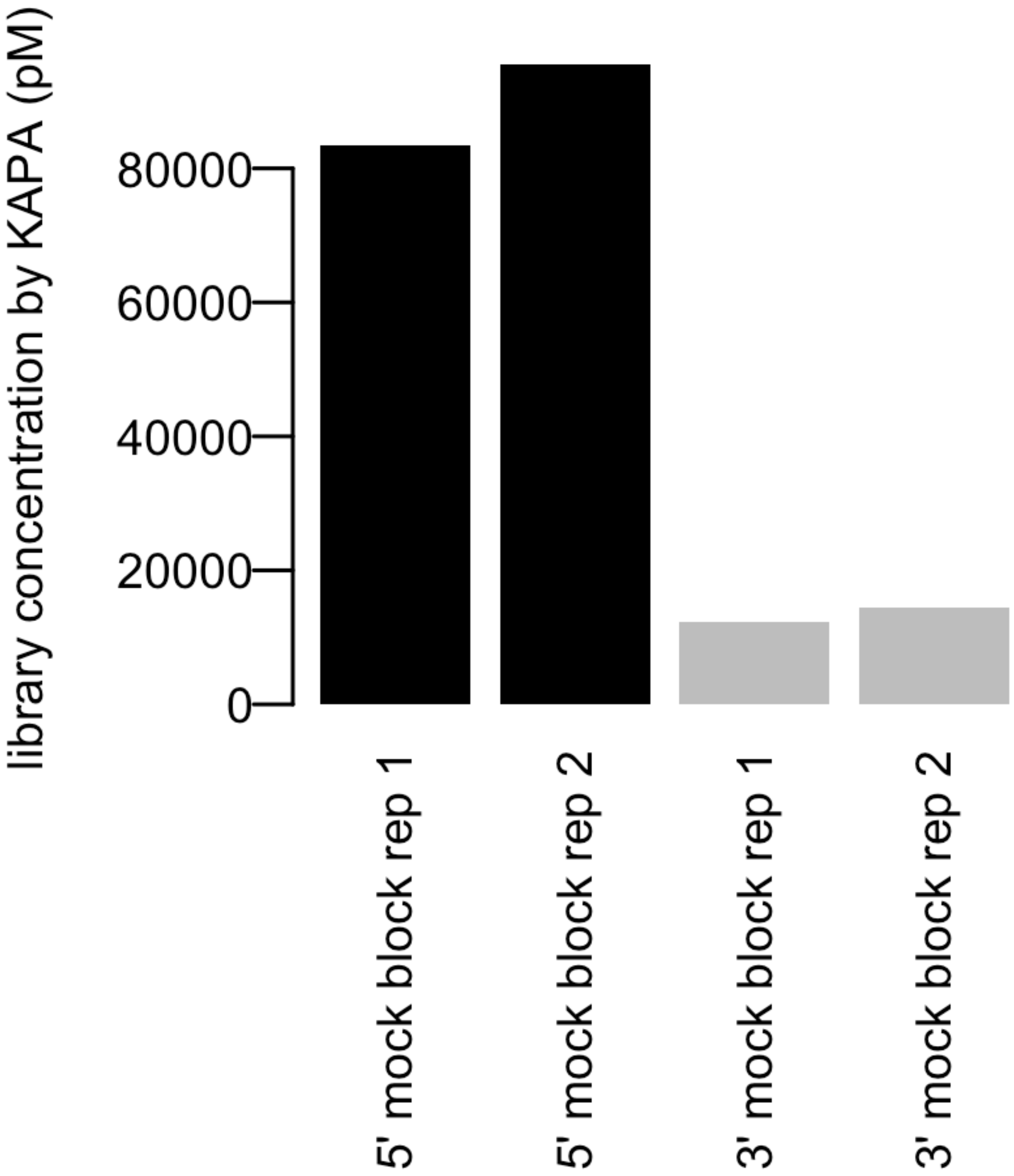
library concentration by KAPA (pM)
80000
60000
40000
20000
0
5' mock block rep 1
5' mock block rep 2
3' mock block rep 1
3' mock block rep 2

Sup. Fig. 3

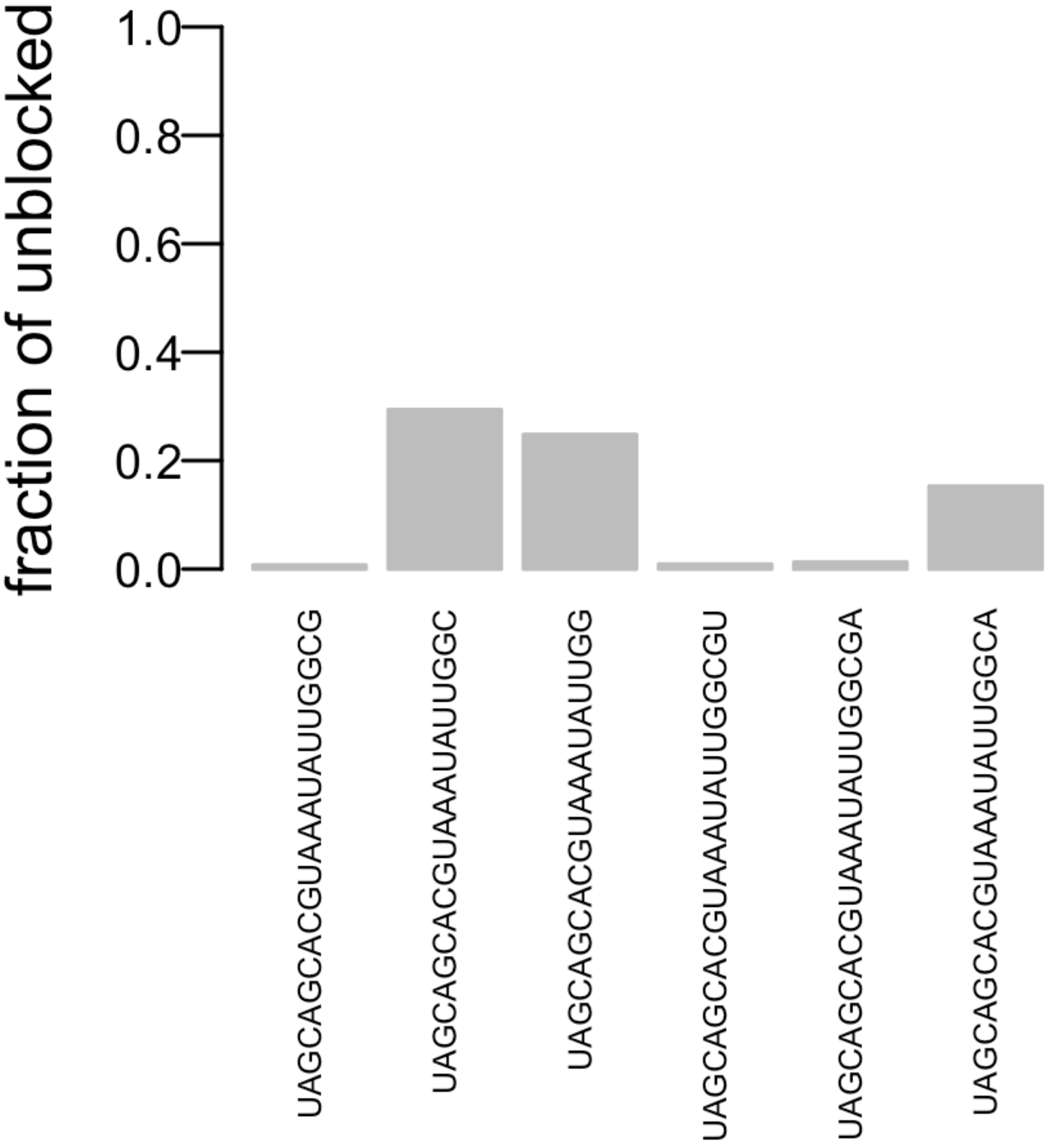
fraction of unblocked
1.0
0.8
0.6
0.4
0.2
0.0
UAGCAGCACGUAAAUAUUGGCG
UAGCAGCACGUAAAUAUUGGC
UAGCAGCACGUAAAUAUUGG
UAGCAGCACGUAAAUAUUGGCGU
UAGCAGCACGUAAAUAUUGGCGA
UAGCAGCACGUAAAUAUUGGCA

Sup. Fig. 4

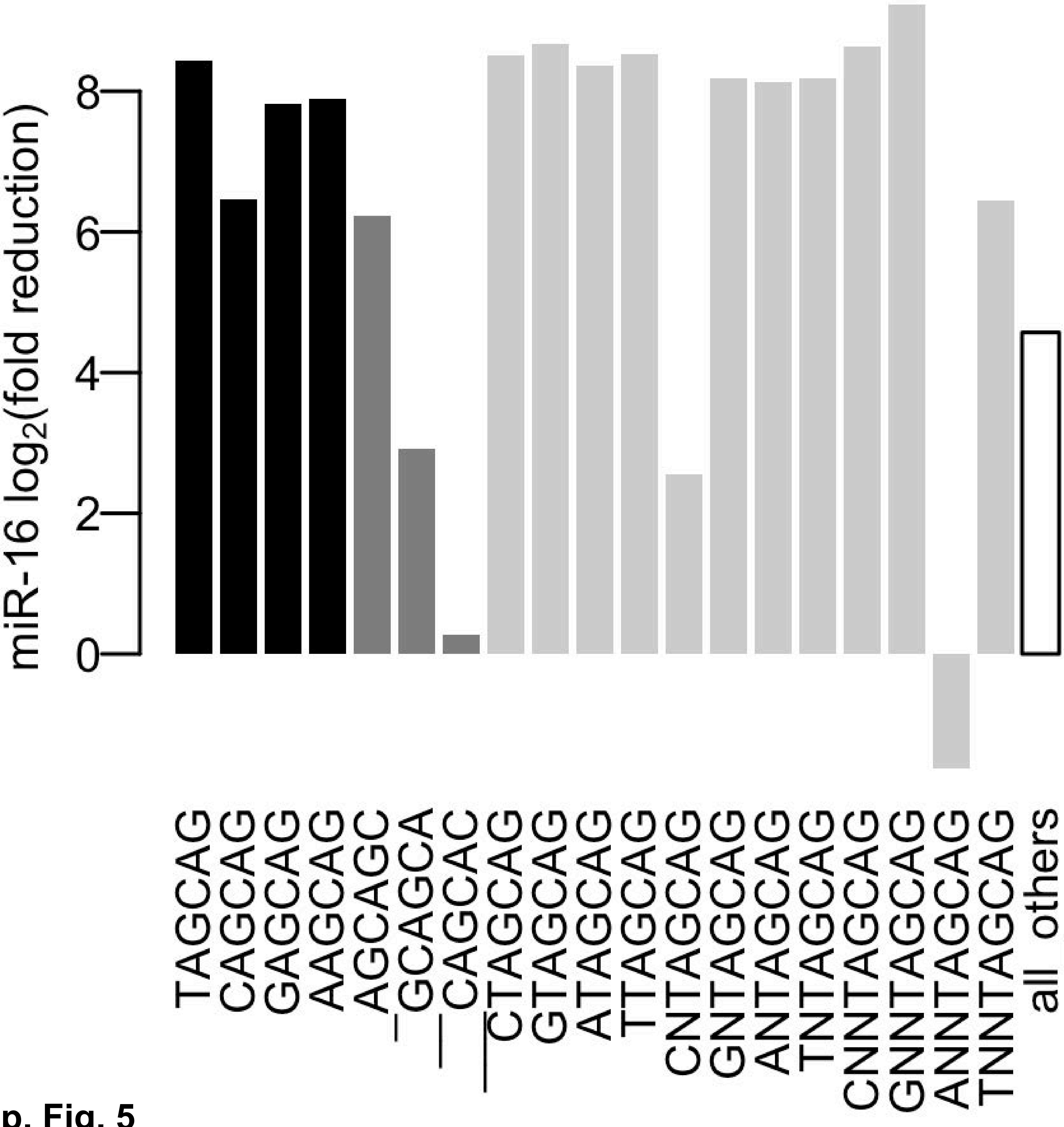

miR-16 log2(fold reduction)
0
2
4
6
8
TAGCAG
CAGCAG
GAGCAG
AAGCAG
AGCAGC
_GCAGCA
_CAGCAC
_CTAGCAG
GTAGCAG
ATAGCAG
TTAGCAG
CNTAGCAG
GNTAGCAG
ANTAGCAG
TNTAGCAG
CNNTAGCAG
GNNTAGCAG
ANNTAGCAG
TNNTAGCAG
all_others

Sup. Fig. 5

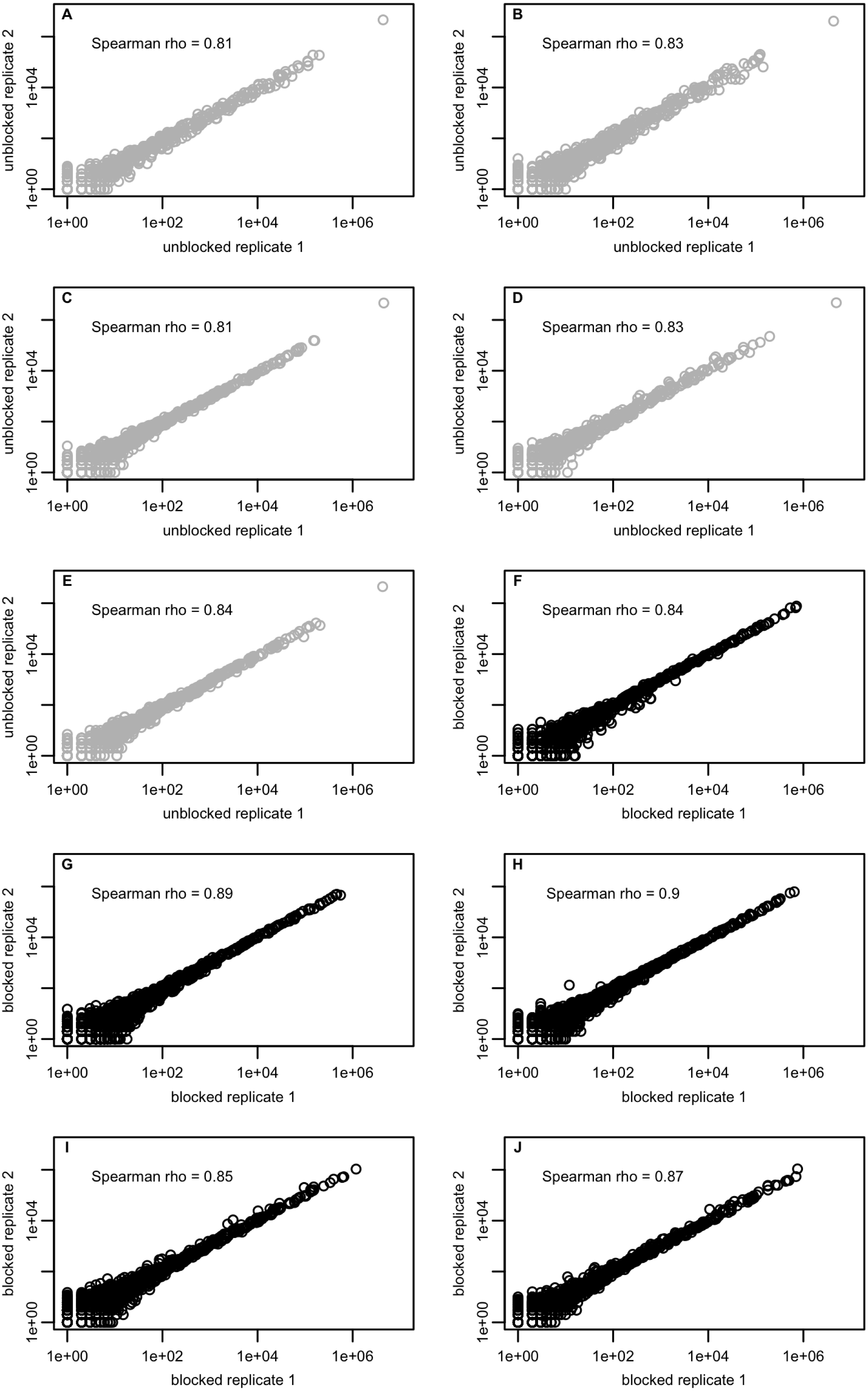
A
Spearman rho = 0.81
unblocked replicate 2
unblocked replicate 1
B
Spearman rho = 0.83
C
Spearman rho = 0.81
D
Spearman rho = 0.83
E
Spearman rho = 0.84
F
Spearman rho = 0.84
blocked replicate 2
blocked replicate 1
G
Spearman rho = 0.89
H
Spearman rho = 0.9
I
Spearman rho = 0.85
J
Spearman rho = 0.87
1e+00
1e+02
1e+04
1e+06

Sup. Fig. 6

Supplementary Figure S1. Categorical distribution of reads from a set of unblocked human plasma libraries. The fraction of reads falling into six categories for 27 libraries derived from human plasma samples is shown. Blue indicates reads aligning to miRNAs but not to hsa-miR-16-5p. Purple indicates reads that map to the human genome but are not miRNAs. Red indicates reads aligning to hsa-miR-16-5p. Green indicates reads that align to the spike-ins. Black are reads that failed to align to miRNAs or the human genome. Gray are reads that are adaptor-dimer.

Supplementary Figure S2. Categorical distribution of reads from a set of hsa-miR-16-5p blocked human plasma libraries. The fraction of reads falling into six categories for 23 libraries derived from human plasma samples in which hsa-miR-16-5p was blocked is shown. Blue indicates reads aligning to miRNAs but not to hsa-miR-16-5p. Purple indicates reads that map to the human genome but are not miRNAs. Red indicates reads aligning to hsa-miR-16-5p. Green indicates reads that align to the spike-ins. Black are reads that failed to align to miRNAs or the human genome. Gray are reads that are adaptor-dimer.

Supplementary Figure S3. Effect of the blocking ligation reaction when targeting the 5' end versus targeting the 3' end. Plotted is the total library concentration as determined using the Library Quantification Kit - Illumina/ABI Prism (KAPA Biosystems). The black bars are libraries in which a mock blocking ligation (all reagents except the blocking oligonucleotide) was run as would be performed to block the 5' end of a targeted miRNA. The gray bars are libraries in which a mock blocking ligation was run as would be performed to block the 3' end of a targeted miRNA.

Supplementary Figure S4. 3' end variations in hsa-miR-16-5p effects on blocking efficacy by a blocker targeting the 3' end. Shown are various sequence variants of hsa-miR-16-5p, with the canonical form displayed as the leftmost sequence. Together, the six plotted here comprise over 91% of the sequences aligning to hsa-miR-16-5p in this experiment. The bar height indicates the fraction remaining in the blocked library when compared to the unblocked library.

Supplementary Figure S5. 5' end variations in hsa-miR-16-5p effects on blocking efficacy by a blocker targeting the 5' end. Shown are various sequence variants of hsa-miR-16-5p, with the canonical form displayed as the leftmost sequence. Underscores represent "missing" bases from the canonical form. Variants shorter than the canonical form, and certain longer forms show decreased blocking efficiency. Because these variants represent a very small fraction of the total reads (<2%), it is unclear if the base calls represent true variants or sequencing errors.

Supplementary Figure S6. Reproducibility of read counts in libraries with and without hsa-miR-16-5p blocking. (**A-E**) Read counts for replicate unblocked libraries from five human plasma samples are plotted versus each other. (**F-J**) Read counts for replicate hsa-miR-16-5p blocked libraries from five human plasma samples are plotted versus each other. For all experiments, the aligned reads were down-sampled to 6 million before plotting. The Spearman rho coefficient of correlation is shown for each replicate pair.

Supplementary Methods for Roberts et al.

Library Generation Protocol with Blocking

This protocol is a modification of the method described in:
"Barcoding bias in high-throughput multiplex sequencing of miRNA." Alon S. et al. Genome Res. 2011 Sep;21(9):1506-11. Epub 2011 Jul 12.

Overview

This protocol describes the preparation of multiplexed (barcoded) libraries of miRNA from total RNA samples suitable for sequencing on the Illumina HiSeq and GAII platforms. The total RNA must be prepared by a technique that captures short RNA species (15nt-25nt). Acceptable techniques are phenol-chloroform extraction followed by ethanol precipitation or NorGen columns, amongst others.

MicroRNA species in the samples have an adaptor oligo (referred to as the 3' adaptor) ligated to their 3' ends. Next a different oligo (referred to as the 5' adaptor) is ligated to the 5' end. The 3' adaptor provides a binding site for a complementary RT primer. This allows for cDNA to be made from the miRNA-adaptor complex via reverse transcription. The cDNA is then used as a template for several rounds of PCR. The PCR primers have long tails (~30 nt) that extend the length of the product. The tails contain the barcoding sequences (with an index read primer binding site), the Illumina sequencing primer site, and the Illumina cluster-generating sequences.

Considerations

The first step, the 3' ligation, is probably the most important step in the protocol. It hinges on the use of a truncated form of T4 RNA ligase. The truncation renders the enzyme unable to use ATP for energy and instead must use an already adenylated oligo as a substrate. Consequently, the 3' adaptor oligo is adenylated on its 3' end. If a fully functional RNA ligase capable of using ATP were used in this step, it would ligate the various RNA species present into concatomers, instead of only ligating the adaptor to the target RNA species. However, one must appreciate that all RNA species are targets, not just the miRNAs, leading to the formation of numerous unintended ligation products. Not only must these products be removed prior to sequencing, the other RNA species distract the adaptor from the miRNA population. Although it is difficult to calculate directly, the effective efficiency of ligation of the miRNAs present, in terms of the percent of miRNAs that actually get ligated to a 3' adaptor, is likely low. The 3' adaptor also has a 3-carbon spacer on its 5' end. This is to prevent RNA from being ligating to its 5' end in the subsequent 5' ligation reaction, which uses full length T4 RNA ligase.

Although the unintended ligation products that occur when an RNA molecule present in the sample other than a miRNA is ligated are somewhat problematic, the most problematic product formed in this protocol arises when un-ligated 3' adaptor is ligated to the 5' adaptor creating an adaptor dimer in the second ligation step. This creates a short product that following PCR is highly complementary to the intended miRNA ligation product. In fact, they only differ by the internal ~22 bp of the miRNA. The adaptor dimer will hybridize efficiently to the intended miRNA product, making the separation of the two difficult. This problem is somewhat helped by hybridizing the RT primer to the 3' ligation product. Since the RT primer is complementary to the entire length of the 3' adaptor, the hybridization serves to bind some of the un-ligated 3' adaptor, preventing adaptor dimer formation in the 5' ligation reaction. While this technique reduces the formation of the adaptor dimer, much still persists and is present after PCR. It must be separated from the intended product by gel electrophoresis. However, because of the strong hybridization between the adaptor dimer and the intended miRNA product, the gel must be run under extremely denaturing conditions. To accomplish this, 10% acrylamide TBE-Urea gels are used. Furthermore, the gels are run in pre-heated buffer (90C). Although it is inconvenient to run the hot gels, our studies have shown that the 10% acrylamide TBE-Urea gels run at room temperature are not sufficiently denaturing for this application.

Detailed Procedure

<u>General Notes</u>

For those steps in which multiple components are added to the reaction, best practice is to make a "master mix" of the components sufficient for all reactions being performed. The samples throughout the course of the protocol should always be kept on ice or at ice temperature when not being otherwise incubated. In the development of this protocol, the samples were kept in a metal block that was kept cool in a refrigerator when not in use. (For simplicity, the protocol will say "on ice", however.) Furthermore, the T4 RNA Ligase 2, truncated; the RNAse Inhibitor, murine; the T4 RNA Ligase 1, the SuperScript II, and the Phusion PCR Master Mix should all be kept on ice. In the following step, "**STOPPING POINT**" is written at points where the protocol can be stopped overnight. This protocol can be completed in 3 days. If the precipitation overnight incubations at -30C are shortened to 2 hour incubations at -80C, the protocol can be done in 2 busy days.

<u>3' Ligation</u>

1. Prepare the following stock buffer, called "2X 3' Ligation Buffer". This recipe is sufficient for many reactions and does not need to be prepared fresh each time the protocol is run. Store at -20C between uses:

   250 uL 50% PEG 8000 (from T4 RNA Ligase 1 kit)
   200 uL 10X T4 RNA Ligase Buffer (from T4 RNA Ligase 1 kit)

550 uL DNAse, RNAse free water

2. Make a stock solution of the spike-in controls. Make a large batch suitable for multiple runs of this protocol. Store at -80C. The concentrations listed here are suitable for human plasma samples. However, it is expected that the total input of the spike-ins will need to be adjusted for different sample types.

   Final Concentrations:
   20 pM miRNASeq Multiplex 22bp Spike In
   2 pM miRNASeq Multiplex 25bp Spike In
   0.2 pM miRNASeq Multiplex 20bp Spike In

3. Combine the following in a 0.2 mL PCR tube.

   1 uL 10uM miRNASeq Multiplex 3' Adaptor
   1 uL Spike In stock (from step 2)
   4 uL of total RNA

4. Gently mix by flicking the tube and spin down the tube in a tabletop mini-centrifuge. Incubate for 2 min at 70C in a pre-heated thermal cycler. Immediately chill on ice following incubation.

5. To each sample add the following:

   10 uL 2X 3' Ligation Buffer
   2 uL T4 RNA Ligase 2, truncated
   1 uL RNAse Inhibitor, murine

6. Gently mix the components and spin down. Incubate for 1 hour at 25C in a thermal cycler. (Note: Incubation times longer than 1 hour have been shown to produce undesired products.)

<u>Blocking Ligation</u>

1. Pre-anneal the blocking oligonucleotide (do this every time). Incubate a 0.5 uM blocking oligonucleotide stock in 1X T4 DNA Ligase buffer as follows:

   95C for 5 min
   65C for 5 min
   55C for 5 min
   45C for 5 min
   35C for 5 min
   25C for 5 min
   4C for infinity

2. Make a master mix of the following:

1 uL of pre-annealed blocking oligonucleotide working stock
1 uL of 10 mM ATP
1 uL of T4 DNA Ligase

3. Add 3 uL of the above master mix to each 3' ligation reaction.
4. Incubate at 30C for 1 hr followed by 65C for 10 min and hold at 4C.

RT Primer Hybridization, and 5' Ligation

1. To the 3' ligation product, add 1uL of 10 uM miRNASeq Multiplex RT Primer. Incubate as follows in a thermal cycler:

   75C for 5 min
   37C for 30 min
   25C for 15 min
   4C for inf

2. While the samples are incubating, thaw the 20 uM miRNASeq Multiplex 5' Adaptor. Once thawed, incubate the adaptor at 70C for 2 min and then immediately chill on ice.

3. A pool of 4 5' adaptors is used in the next step. These are a equimolar mix of miRNASeq Multiplex 5' Adaptor Mod 1, 2, 3, and 4 at 5uM final concentration each, for a total adaptor concentration of 20 uM.

4. When the samples are finished incubating, transfer them to ice. Add the following:

   0.64 uL T4 RNA Ligase 1
   1 uL RNAse Inhibitor, murine
   0.86 uL RNAse , DNAse free water
   1 uL 20 uM miRNASeq Multiplex 5' Adaptor Mod pool
   1 uL 10X T4 RNA Ligase Buffer (T4 RNA Ligase 1 kit)
   1 uL 10 mM ATP (T4 RNA Ligase 1 kit)

5. Mix gently and spin down briefly. Incubate the samples for 1 hour at 25C in a thermal cycler. **STOPPING POINT** (The samples can be placed in -80C and left overnight after this step, although it is ideal to take the samples through reverse transcription before stopping)

Reverse Transcription and PCR

1. Setup the following reaction. The protocol up this point has generated ~ 26.5 uL of ligated product. Only 11 uL of the product is carried forward, so that

the remainder is available for a repeat if needed. The unused product should be stored at -80C.

4 uL 5X FS Buffer (SuperScript II kit)
2 uL 0.1 M DTT (SuperScript II kit)
1 uL Deoxynucleotide Mix (10 mM each)
1 uL RNAse Inhibitor, murine
1 uL SuperScript II (SuperScript II kit)
11 uL ligation product (from previous step)

2. Incubate the samples in a thermal cycler as follows:

   42C for 50 min
   70C for 15 min
   4C for inf

3. Add the following to each sample:

   25 uL Phusion High-Fidelity PCR Master Mix
   2.5 uL 20 uM miRNASeq Multiplex R Primer

   To each individual sample add 2.5 uL of one of the twelve different indexed miRNASeq Multiplex F Primers at 20 uM, being sure to note which sample received which barcoded primer. Mix the samples and spin down.

4. Incubate the samples in a thermal cycler as follows:

   94C for 30s

   15 cycles of:
   94C for 10s
   72C for 45s

   65C for 5 min
   4C for inf

   STOPPING POINT (The samples can be stored at -20C)

Concentration, Gel Separation, and Purification

The gels run in this protocol are the Mini-PROTEAN format from BioRad and run in the Mini-PROTEAN Tetra Cell gel system. It is expected that using a different gel system would require that extensive modifications be made to this protocol.

1. Transfer each sample to a 1.7 mL microcentrifuge tube. Add 250 uL of Buffer PB (MinElute Kit). Mix well and transfer to a MinElute column placed in a

2mL collection tube. Centrifuge for 1 min at max speed. Discard flow through.

2. Add 750 uL of Buffer PE (MinElute kit, ensure ethanol has been added) to the MinElute column. Centrifuge for 1 min at max speed. Discard flow through and place column back into the same collection tube. Centrifuge again for 1 min at max speed.

3. Transfer the column to a clean 1.7 mL microcentrifuge tube. Add 17.5 uL of RNAse, DNAse free water. Let stand for 5 min. Centrifuge for 1 min at max speed. Discard column, keeping the flow through in the microcentrifuge tube.

4. To each sample, add 17.5 uL of 2X TBE-Urea Sample Buffer. Mix well and spin down. Set the samples aside at room temperature.

5. Prepare DNA ladder working solutions. This recipe makes enough for several runs and need not be made fresh. Store at 4C.

   <u>20 bp Ladder</u>
   200 uL 2X TBE-Urea Sample Buffer
   180 uL DNAse, RNAse free water
   20 uL 20 bp DNA Ladder stock solution (Bayou BioLabs)

   <u>100 bp Ladder</u>
   200 uL 2X TBE-Urea Sample Buffer
   190 uL DNAse, RNAse free water
   10 uL 100 bp DNA Ladder stock solution (NEB)

6. At this point in the protocol, a hot gel will be run. Since this involves using near-boiling TBE buffer, extreme caution should be used. Additionally, protective equipment such as aprons and gloves should be worn.

7. Preheat a heating block to 95C.

8. Make 1X TBE buffer from 10X TBE buffer stock. Make 1 liter, sufficient for one or two gels. A single gel can accommodate four samples with no spacer lane between samples. Each sample will be split and run in two lanes to avoid interference from the adaptor dimer. It is not recommended to run more than two gels at a time.

9. Pre-warm 10% TBE-Urea Mini-PROTEAN gel(s) in hot tap water (no hotter than what comes out of the tap). Leave them in their packaging and weigh them down so they don't float. Also, warm the gel holder in the water.

10. In a microwave, heat 900 mL of 1X TBE buffer split into aliquots of 450 mL in two 500 mL Pyrex beakers with Saran wrap partially covering the top to 80C-85C. Heat in increments of 2-5 min (depending on microwave power). Between heating increments, carefully stir the buffer with a thermometer and check the temperature. Do not boil the buffer.

11. When the heating of the buffer is nearing completion, place the samples into the preheated heating block at 95C. Also place the 20 bp and 100 bp working solutions in the heating block. Ensure that every sample resides at 95C for at least two minutes before it is loaded onto the gel. It is not detrimental for the samples to remain in the heating block for more than 2 min, up to ~30 min.

12. Remove the gels and gel holder from the warm water. Remove the gels from their packaging, ensuring to remove the green tape at the bottom of the gel and the lane comb. Assemble the gels in the gel holder.

13. Pour the now hot 1X TBE buffer (80C-85C) into the gel assembly, filling it to the top.

14. With a p20 set to 15 uL, pipet up and down in each well of the gel. This is to remove any urea that often crystalizes in the wells during storage. Remove any bubbles in the wells.

15. Remove the two ladder tubes (carefully, they are hot). Spin them down briefly in a tabletop mini centrifuge. Add 15 uL of the 20 bp ladder to lane 1 of the gel, pipetting carefully to avoid contamination of other lanes. The tube may make a "pop" when opened. Add 15 uL of the 100 bp ladder to lane 2.

16. Remove a pair of sample tubes from the heating block. Spin them down briefly in a tabletop mini centrifuge. Load two 15 uL aliquots of each sample into two adjacent lanes of the gel. Repeat for all of the samples. Work quickly because the gel is cooling, but carefully and deliberately.

17. Once all the samples are loaded, gently place the gel assembly into the gel box. Re-heat the remaining 1X TBE buffer to 90C in the microwave. Pour all the remaining 1X TBE into the gel box (not inside the gel assembly).

18. With a 10 mL pipet, top-off the buffer inside of the gel assembly with buffer in the gel box, filling it as near to the top as possible. This is important because the hot buffer will evaporate during the course of the run.

19. Begin running the gel at 200V. Closely monitor the current. If the current begins to rise more than 10 mA from the initial current (this is likely to happen), turn the voltage down 10V to 190V. Continue to monitor the current and adjust the voltage lower until the current stabilizes. However, do not run the gel below 160V. The current rises because the gel and buffer

are hot. The conductivity of the system is much higher than when run at room temperature. The increased conductivity allows more current to flow, which in turns heats the gel, further increasing conductivity, and creating a positive feedback loop. Thus, the current must be monitored closely during the run. Under these conditions, the gel should be run for 45 minutes.

20. Turn off the power source and disassemble the gel box. Allow the gels to cool on the bench top prior to opening their plastic cases. While the gels are cooling, for each gel, add 50 mL of 1X TBE to a suitably sized gel staining container. Add 5 uL of SYBR Gold 10,000X stock to each 50 mL TBE aliquot and mix. Wrap the container in aluminum foil to protect it from light. Open the plastic case of the now cooled gel and place the gel into the staining container with the TBE and SYBR Gold. Re-cover the container with the aluminum foil and rock on a gel rocker for 10 minutes.

21. While the gel is staining, prepare the following for each sample. With a 20-gauge needle, poke a hole in the bottom of a 0.5 mL microcentrifuge tube. Place this tube into a 1.7 mL centrifuge tube.

22. Place a sheet of Saran wrap on a UV-transilluminator. Transfer the gel from the staining solution onto the Saran wrap sheet. Capture an image of the gel under UV illumination with an appropriate gel visualization system (i.e. UVP EC3 Imaging System).

23. Transfer the gel by picking up the Saran wrap to a UV-transilluminator that can be accessed for subsequent gel excision steps (maybe the same as where the image was taken). With razor blades and forceps, carefully excise the 135 bp band for each sample (see Figure 1). Since each sample was loaded in two aliquots in adjacent lanes, cut both bands from the same sample out together. Replace the razor blades and forceps after every time they touch the gel to avoid cross-contamination. Place the gel fragments into the 0.5 mL microcentrifuge tube with the hole in the bottom.

24. Transfer the 0.5 mL microcentrifuge tubes nested in 1.7 mL microcentrifuge tubes containing the gel pieces into a microcentrifuge. Spin at max speed for 1 min. The gel fragment should be in the bottom of 1.7 mL microcentrifuge tube in small pieces. If some of the gel fragment is retained in the 0.5 mL, spin at max speed for another minute.

25. Prepare the following stock, called "Soaking Solution". This recipe makes enough for many samples as does not need to be prepared fresh every time. Store at room temperature.

    2 mL 5M Ammonium Acetate
    2 mL 1% SDS solution
    4 uL 0.5M EDTA

16 mL RNAse, DNAse free water

26. Add 300 uL of the Soaking Solution to each sample. Incubate with agitation at 70C for 2 hours.

27. Transfer each sample (including gel pieces) to a Spin-X Centrifuge Tube Filter, 0.22 um Cellulose Acetate, sitting in its accompanying microcentrifuge tube. Spin in a microcentrifuge at max speed for 1 min.

28. Transfer the flow-though to a new 1.7 mL microcentrifuge tube. Add 1 uL of 10 ug/uL glycogen. Add 300 uL of 100% isopropanol. Vortex and spin down briefly. Incubate overnight at -30C. **STOPPING POINT** (The samples can be kept in the precipitating conditions at -30C for several days.)

29. Spin the samples in a refrigerated centrifuge (4C) for 20 min at 14,000 rpm (max speed). Again, place the hinges of the tubes outward so that the location of the pellet in predictable.

30. While the samples are in the centrifuge, chill an aliquot of 80% ethanol by place in it in ice water or by some other suitable method.

31. After centrifugation, pipet off the supernatant. Using a p200, place the tip of the pipet near the bottom of the tube away from the hinge side and gently remove the liquid. Add 100 uL of the chilled 80% ethanol and centrifuge again in a refrigerated centrifuge (4C) for 10 min at 14,000 rpm (max speed).

32. Again carefully remove the supernatant with the p200 as described above. After removing as much as possible with the p200, use a p20 to get the remainder, leaving behind as little liquid as possible.

33. Resuspend the pellet in 10 uL of EB buffer (MinElute Kit). Measure the concentration of the sample with a suitable method (QBit HS DNA is preferred with 1uL of sample input). The sample is ready for sequencing. Typically, this protocol yields 10 uL of 1-4 ng/uL product, depending on sample input mass and sample type. Although it depends on the level of multiplexing, 0.5 ng/uL or higher libraries are concentrated enough for sequencing. If a lower yield is expected, the pellet can be resuspended in a lower volume to yield a higher concentration product.

34. It is highly recommended to run KAPA qPCR to quantify library concentrations before sequencing.

Table 1 - Oligo List

Name	Seqeunce
miRNASeq Multiplex 3' Adaptor	/5rApp/ACGGGCTAATATTTATCGGTGG/3SpC3/
miRNASeq Multiplex 5' Adaptor Mod 1	rUrCrCrCrUrArCrArCrGrArCrGrCrUrCrUrUrCrCrGrArUrCrUrCrArGrUrCrG
miRNASeq Multiplex 5' Adaptor Mod 2	rUrCrCrCrUrArCrArCrGrArCrGrCrUrCrUrUrCrCrGrArUrCrUrUrGrArCrUrC
miRNASeq Multiplex 5' Adaptor Mod 3	rUrCrCrCrUrArCrArCrGrArCrGrCrUrCrUrUrCrCrGrArUrCrUrGrCrUrArGrA
miRNASeq Multiplex 5' Adaptor Mod 4	rUrCrCrCrUrArCrArCrGrArCrGrCrUrCrUrUrCrCrGrArUrCrUrArUrCrGrArU
miRNASeq Multiplex RT primer	GCTCCACCGATAAATATTAGCCCGT
miRNASeq Multiplex 22bp Spike In	rArGrCrGrCrUrUrGrCrArGrArGrArGrArArArUrCrArG
miRNASeq Multiplex 25bp Spike In	rGrCrGrUrGrGrArCrArCrArUrCrUrGrUrCrGrGrCrCrArUrArC
miRNASeq Multiplex 20bp Spike In	rArArCrCrGrCrArCrArArCrCrUrGrCrCrGrArUrG
Hsa-miR-16-5p Blocker	/5pC3/TACGTGCTGCTACGTACTCTGGACTCTAGTCAGTAGCACGACTAGAGTCCAGAGTACG
Hsa-miR-451a Blocker	/5SpC3/TGGTAACGGTTTCGTACTCTGGACTCTAGTCAGTAGCACGACTAGAGTCCAGAGTACG

PCR Primers	Sequence	Barcode
miRNASeq Multiplex R Primer	AATGATACGGCGACCACCGAGATCTACACTCTTTCCCTACACGACGCTCTTCCGATCT	
miRNASeq Multiplex F Primer Index 1	CAAGCAGAAGACGGCATACGAGATCGTGATGCTCCACCGATAAATATTAGCCCGT	ATCACG
miRNASeq Multiplex F Primer Index 2	CAAGCAGAAGACGGCATACGAGATACATCGGCTCCACCGATAAATATTAGCCCGT	CGATGT
miRNASeq Multiplex F Primer Index	CAAGCAGAAGACGGCATACGAGATGCCTAAGCTCCACCGATAAATATTAGCCCGT	TTAGGC

3		
miRNASeq Multiplex F Primer Index 4	CAAGCAGAAGACGGCATACGAGATTGGTCAGCTCCACCGATAAATATTAGCCCGT	TGACCA
miRNASeq Multiplex F Primer Index 5	CAAGCAGAAGACGGCATACGAGATCACTGTGCTCCACCGATAAATATTAGCCCGT	ACAGTG
miRNASeq Multiplex F Primer Index 6	CAAGCAGAAGACGGCATACGAGATATTGGCGCTCCACCGATAAATATTAGCCCGT	GCCAAT
miRNASeq Multiplex F Primer Index 7	CAAGCAGAAGACGGCATACGAGATGATCTGGCTCCACCGATAAATATTAGCCCGT	CAGATC
miRNASeq Multiplex F Primer Index 8	CAAGCAGAAGACGGCATACGAGATTCAAGTGCTCCACCGATAAATATTAGCCCGT	ACTTGA
miRNASeq Multiplex F Primer Index 9	CAAGCAGAAGACGGCATACGAGATCTGATCGCTCCACCGATAAATATTAGCCCGT	GATCAG
miRNASeq Multiplex F Primer Index 10	CAAGCAGAAGACGGCATACGAGATAAGCTAGCTCCACCGATAAATATTAGCCCGT	TAGCTT
miRNASeq Multiplex F Primer Index 11	CAAGCAGAAGACGGCATACGAGATGTAGCCGCTCCACCGATAAATATTAGCCCGT	GGCTAC
miRNASeq Multiplex F Primer Index 12	CAAGCAGAAGACGGCATACGAGATTACAAGGCTCCACCGATAAATATTAGCCCGT	CTTGTA

Table 2 - Materials

Product Name	Component	Manufacturer	Cat #
T4 RNA Ligase 2, truncated	T4 RNA Ligase 2, truncated	NEB	M0242L
RNAse Inhibitor, Murine	RNAse Inhibitor, Murine	NEB	M0314S
T4 RNA Ligase 1	T4 RNA Ligase 1	NEB	M0204S
T4 RNA Ligase 1	10X T4 RNA Ligase Reaction Buffer	NEB	M0204S
T4 RNA Ligase 1	10 mM ATP	NEB	M0204S
T4 RNA Ligase 1	50% PEG 8000	NEB	M0204S
T4 DNA Ligase	T4 DNA Ligase	NEB	M0202M
T4 DNA Ligase	10X T4 DNA Ligase Buffer	NEB	M0202M
SuperScript II Reverse Transcriptase	SuperScript II RT	Invitrogen	18064-071
SuperScript II Reverse Transcriptase	5X FS Buffer	Invitrogen	18064-071
SuperScript II Reverse Transcriptase	0.1 M DTT	Invitrogen	18064-071
Deoxynucleotide Solution Mix	Deoxynucleotide Solution Mix	NEB	N0447S
Phusion High-Fidelity PCR Master Mix with HF Buffer	Phusion High-Fidelity PCR Master Mix with HF Buffer	NEB	M0531S
Ethanol	Ethanol	Sigma-Aldritch	E7023-500ML
MinElute PCR Purification Kit	MinElute Spin Column	Qiagen	28004
MinElute PCR Purification Kit	Buffer PB	Qiagen	28004
MinElute PCR Purification Kit	Buffer PE (concentrate)	Qiagen	28004
MinElute PCR Purification Kit	Buffer EB	Qiagen	28004
Sodium Acetate, 3M, molecular biology grade	Sodium Acetate, 3M, molecular biology grade	Usb	75897
100 bp DNA Ladder	100 bp DNA Ladder	NEB	N3231L
20 bp DNA Ladder	20 bp DNA Ladder	Bayou BioLabs	L-100
TBE-Urea Sample Buffer	TBE-Urea Sample Buffer	BioRad	161-0768
10% Mini-PROTEAN TBE-Urea Precast Gel	10% Mini-PROTEAN TBE-Urea Precast Gel	BioRad	456-6033
Accugene 10X TBE Buffer	Accugene 10X TBE Buffer	Lonza	50843
SYBR Gold 10,000X	SYBR Gold 10,000X	Invitrogen	S11494
5M Ammonium Acetate	5M Ammonium Acetate	Ambion	AM9070G
0.5M EDTA	0.5M EDTA	Ambion	AM9261
Sodium Dodecyl Sulfate (SDS)	Sodium Dodecyl Sulfate (SDS)	Sigma-Aldritch	L6026-50G
Spin-X Centrifuge Tube Filter	Spin-X Centrifuge Tube Filter	Costar	8161
Glycogn 20ug/uL	Glycogn 20ug/uL	Invitrogen	10814-010
Library Quantification Kit - Illumina/ABI Prism	Library Quantification Kit - Illumina/ABI Prism	KAPA Biosystems	KK4835
Isopropanol, Molecular Biology Grade	Isopropanol, Molecular Biology Grade	Fisher	BP2618-500
